# Saudi Medical Journal 2022

**DOI:** 10.15537/smj.2023.44.1.20230016

**Published:** 2023-01

**Authors:** Fahdah Alokaily

The pandemic has affected public health and scientific publication significantly, there was a dramatic rise in COVID-19 publications associated w-ith a substantial decrease in non-COVID-19 research. Similar to last year, 25% of the papers published in Saudi Medical Journal (SMJ) were Covid-related articles; and 75% of which constitutes local contributions. We do encourage contributions from other parts of the globe to continue advancing the knowledge on Covid-19 related issues.

We hope in 2023 that the dark cloud of COVID-19 is fading away with the present implementation of laws, restrictions, and public health education.

Some publishing trends have emerged and become popular, especially during the start of the pandemic, one of them is publishing on preprint servers especially since Crossref began assigning DOIs to preprint in 2016.^1^ Authors seek quick feedback, fast dissemination, citation, and the primacy of their work so they prefer to submit their preliminary, non-peer-reviewed articles to these repositories than the traditional journals. Submission to preprint servers is a personal publishing choice of the author, the peer review process improves but does not eliminate incomplete reporting and spin, however, we at Saudi Medical Journal do not consider submissions of manuscripts posted in preprint servers and also do not consider the use of preprint references. Thus, we revised our policy when it comes to articles that were submitted to these repositories. Any material that has been publicly available, with a permanent identifier and citable will no longer be considered for submission.^2^

The journal follows the double-blind peer review policy so having a paper posted in preprint servers, publicly available and visible with DOI, with full details of authors such as ORCID will nullify the purpose of the double-blind policy we apply.

In early January 2022, the Saudi Medical Journal faced some technical issues with the submission system that probably affected the submission volume and a slight decline in citations.

Peer review is vital to maintain the integrity of science by critically assessing and validating the originality and scientific merit of the article before publication and dissemination of information, hence, we discourage preprint.

One of the struggles we faced in the peer-review process is keeping the reviewers complete the review of revised versions. Prior to the submission of the review, the reviewers are asked if should they wish to see the article again after it has been revised. Only the reviewers who agreed to see the revised version will be invited and very few reviewers are able to follow this workflow. When a reviewer declines to review the revised version or is not available, this causes lag in the decision process and leaves the editor the task to go over the suggestions of the reviewers. Despite all the technical innovations available, it is still difficult to attract qualified reviewers and establish a dedicated editorial board. Perhaps, a survey for reviewers regarding the evaluation of the revised will help us understand and improve our peer-review process

Other ways of addressing this challenge, Saudi Medical Journal highly recognize the reviewer’s contribution to the peer review process. Apart from publishing their names in the yearly Editorial Message, we have activated Publons in our peer review system. Therefore, whenever a reviewer submits a review, they will be asked if they want to receive recognition on Publons.

Publons is a free platform that creates and verifies researchers’ peer reviews and editorial contributions. It has joined the Web of Science, so it is linked directly to the Web of Science publication and peer review history. Anyone can register via the Web of Science.^3^

This year, Saudi Medical Journal and Neurosciences in collaboration with the Saudi Pediatric Neurology Society conducted a workshop at the “7th Saudi Pediatric Neurology Society Conference & Workshop.” Saudi Medical Journal’s role in this scientific event was to educate participants on how to write a publishable manuscript. Focusing on writing guidelines, peer review process, publishing tips, common reasons for rejection, and avoiding predatory journals. This overview of the publication processes is beneficial for research fellows and residents and all other health researchers.

Saudi Medical Journal pledges to continue providing educational events. As the event was a success and well received by the attendees, SMJ pledge to continue providing educational events.

## Annual statistics

**Figure 1:**
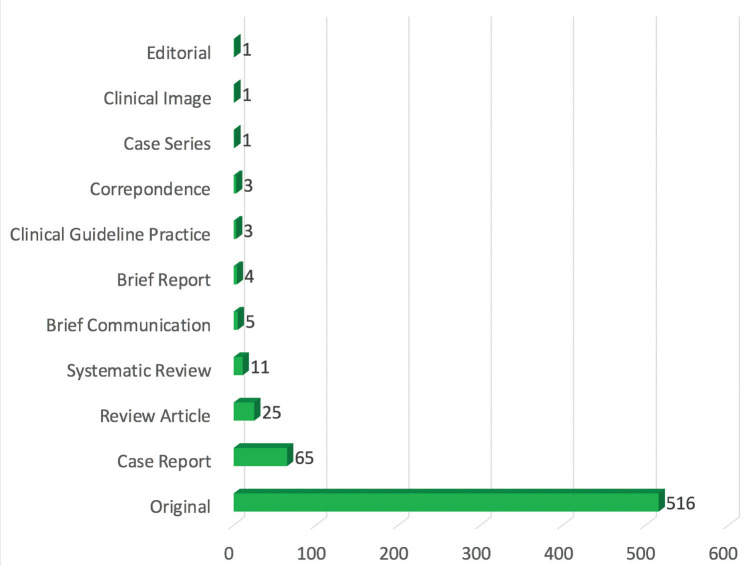
- Type of manuscripts received for the year 2022.

This year we received 635 manuscripts from which we processed 309 articles that have complied with the journal requirements (Figure 1). Over the past 12 issues, we have published one Editorial, 11 Systematic Reviews, and 516 Originals, with a total of 1285 pages. Approximately 78.8% of the papers we published were from the Kingdom of Saudi Arabia (Table 1). Our total rejection rate was 75% of which 51% were rejected at the initial decision. The average processing time frame of original articles in the year 2022 from received date to acceptance was 2.6 months, from acceptance to publication 1.2 months, and from received to publication 3.8 months (Table 2).

**Table 1:** - Origin of “peer-reviewed” articles published in the Saudi Medical Journal for the year 2022.

Origins	n	%
KSA	108	(78.8)
Iraq	3	(2.2)
Iran	1	(0.7)
Egypt	2	(1.5)
Jordan	2	(1.5)
Turkey	23	(16.8)
UK	3	(2.2)
Libya	1	(0.7)
Malaysia	2	(1.5)
Mexico	1	(0.7)
China	12	(8.8)
Bahrain	1	(0.7)
Korea	3	(2.2)
Thailand	1	(0.7)
Indonesia	1	(0.7)
India	2	(1.5)
**Total**	**137**	**(100.0)**

**Table 2:** - Average processing time frame of articles published in the Saudi Medical Journal for the year 2022.

Year	Received to Acceptance (Months)	Acceptance to Publications (Months)	Received to Publications (Months)
2022	2.6	1.2	3.8

## Editorial Message

**Our thanks goes to the reviewers who have participated in the excellent review of manuscripts and books for the year 2022.**

**A.** Seval Ozgu-Erdinc

A.A. Gde Putra Semara Jaya

Abdul Meshikhes

Abdulaziz Mansoor Al Raimi

Abdulbari Bener

Abdulghani Alsaeed*

Abdulla Darwish

Abdullah Abdulkareem

Abdullah Abudayah

Abdullah Alarfaj

Abdullah Alkattan

Abdullah Alkhenizan

Abdullah Awadh

Abdulmoein AlAgha

Abdulmohsen Al-Elq*

Abdulmohsen Al-Zalabani

Abdulrahman Alashkar

Abeer Ali

A. S. Chandrakumari

Abuobaida Abukhelaif*

Adnan Mubaraki

Ahlam El Shikieri

Ahmad Alsaeed

Ahmed Abdel Moneim

Ahmed Absi

Ahmed Al Fagih

Ahmed Al-Ameer

Ahmed Alamri

Ahmed Kadhum

Ahmed Kemalettin Koltka

Ahmed Morad Asaad

Ahmet Aslan

Ahmet Caglayan

Ahmet Kokurcan

Aladdin J. Mohammad

Alberto Modenese

Aleksa Jovanovic

Alexander E. Berezin

Alexandr V. Belousov

Alexandra Senegaglia

Ali A. Alshatwi

Ali Alasmari

Ali Aldhebaib

Ali Alhilly

Ali Alshabi

Ali Alyami

Ali Ismail Ali Abdelreahim

Ali Jawad*

Alian A. Alrasheedy

Altug Tuncel

AM Al-Hinnawi

Amal Ismail

Amari Alhozali

Amjad Alharbi

Amr Arafat

Amro Hanora

Amy Gladin

Anan S. Jarab

Andreas Jahnen

Andreas Tiede

Angham Al Mutair

Anil Kumar Sah

Anirudha Agnihotry

Anna Baran

Anna Horwood

A. M. Badowska-Kozakiewicz

Annang Giri Moelyo

Anne Cusick

Anshoo Malhotra

Anusha Yanamadala

Aqeel Alaqeel

Areej Alkhaldy

Arghya Das

Arife Akay

Arli Aditya Parikesit

Armen Yuri Gasparyan

Ashwin Subramaniam

Asmaa Abdel Nasser

Atousa Deljou

Attia Bari

Aveek Jayant

Aws Al-Numan

Ayman Al-hazmi

Ayse Akbas

**B**adr Aljarallah

Bahar Dernek

Balaji Zacharia

Bander Alanazi

Bang-Gee Hsu

Banu Ordahan

Bareng A.S. Nonyane

Basile N. Landis

Basman Al-Metwali

Bedoor Alomran

Bekir Kocazeybek

Benoit Bedat

Biswanath Basu

**C**arlos Sanchez-Garcia

Carlton Dampier

Cathy L. Lazarus

Cemil Kayali

Chao Li

Charles Doarn

Cheorl-Ho Kim

Christopher J. Nolan

Christos Kosmas

Chuan Shao

Cristina Gluhovschi

Cynthia L. Arfken

**D.** Kafadar

Dan Dupont Hougaard

Daniel Reim

Dara Al-Banna

Dayel Alshahrani

Deependra K. Rai

Derya Karasu

Desh Deepak

D. Kostova-Lefterova

Dhia Kadhem

Dian Handayani

Dina Alsohaibi

Dingshen Lin

Dongsheng Sun

Dragos Ioan Tohanean

**E.** Zangiacomi Martinez

Eiman M. AbdulRahman

Eitaro Kodani

Eman Abdelrazek

Eman Abduljawad

Eman Dyab

Eman E. Elsharkawy

Enas Abdelaziz

Endale Tefera

Erna Rochmawati

**F**adime Kilinc

Fahad Al Juraibah

Fahad Almutairi

F. Mohammadghasemi

Fairoz Ali Al-Wrafy

Faizan Zaffar Kashoo*

Farah Dayana Zahedi

Fares Haddad

Farid Alzhrani

Farrukh Majeed

Faruk Buba

Fatimah Al Aldahan

Fawaz Al-Kassim

Feras Alkholaiwi

Ferdinando Antonio

Gulino

Filiz Ozsoy

Firas S. Azzeh

Foad Abdallah

Fouad Almomani

Francesco Ria

Frederic Triponez

**G**aurav Goyal

Gennaro Martines

Ghada Elgohary

Ghassan Alokby

Giancarlo Micheletto

Gina Joubert

Giovanni Conti

Girmay Fitiwi Lema

Gokhan Ozkan

Grigoris T. Gerotziafas

Gulali Aktas

Gulfem Ece

Gulseren Akyuz

Gunes Senol

Gunnar Hillerdal

**H**. Mazimilian Mehdorn

Hadi Almohsen

Hakan Cokmez

Hakan Turkon

Hala Abdelkarem*

Hala Amer

Halah Mosli

Halil Ozcan

Halls Suleyman

Hamed Adetunji

Hani Mawardi Mawardi

Hao Zhang

Hassan AbdulJabbar

Hassan Abugad

Hassan Al-Trabolsi

Hatice Simsek

Hatim Al-Jifree

Henk Schonewille

Hesham A. AbdelHalim

Hisakazu Nakajima

Hongxun Wu

Hui-shan Wang

**I** Reid

Ibraheem Abosoudah

Ilhan Ece

Ilker M. Arer

Imran Ahmad

Inci Alacacioglu

Isra Aljazeeri

Iyad Naeem Muhammad

**J**acqueline Maria Dias

Jawahir Abuhaimed

Jigeeshu Vasishtha Divatia

Joan L. Robinson

Jone Trovik

Jong Woo Chung

Joshua Barzilay

Jyoshid R. Balan

Jyrki Vuola

**K**azuya Takehana

Kenji Hayashida

Kevin Hellman

Khaled Hamed Salem

Khaled Saad

Khalid Al-Mugheed

Khalid Alshahrani

Khalid Banimelhem

Khalil Abu Jamileh

Kostas Persinakis

**L**. Faggioni*

Lamia Elgarhy

Lamya Alnaim

Lasitha Samarakoon

Latifa Yahya Al Ajeel

Layth Al-Hajjar

Leman Acun Delen

Leo Massari

Liang Zong

Li-ming Yu

Li-Ting Kao

**M**agdalena Baymakova

Magdi Salih

Maha Aleid

Maha Alharthi

Maha Elkholy

Mahwish Faizan

Maja Surbatovic

Mamdouh Allahyani

Mana Almuhaideb

Manar Nabolsi

Man-sang Wong

Marcone Lima Sobreira

Mario Lima

Martin H. Steinberg

Martino Introna

Marwa Abdelhamed

Maryam Al Badi

Maryam Al-Zahrani

Marzia Miglionico

Mateusz Spalek

May Bin-Jumah

Mazen Almehmadi*

Md. Rabiul Islam

Mehmet Ali Eren

Mehmet Artac

Mehmet Burak Eskin

Mert Senturk

Michael D. Crossland

Michele Merli

Milan Lepic

Miltos Lazarides

Mine Kiseli

Mirjana Kostic

Misra Abdulahi

Moba Al Shahed

Mohamed AlAgeel

Mohamed I. Kamel

Mohamed S. Abdel-Latif

Mohammad A. Gaballah

Mohammad Abdelghani

Mohammad Abu Assab

Mohammad Alam

Mohammad Alfuhaily

Mohammad Eid Mahfouz

M. Waheed El-Anwar

Mohammed AlAteeq

Mohammed Alhendi

Mohammed Alsaadi

Mohammed Alsakkaf

Mohammed Salih Mahfouz

Mohd Yazid Bajuri

Mohsen Alzahrani

Mona F. Mahmoud

Morten Dahl

Mostafa Waly

Muath Alammar

Muhammad Kashif Munir

Muhanna AlShaibani

Munierah Al-Hussain

Murat Baykara

Murat Gunay

Mushtak T. S. Al-Ouqaili

Mustafa Arslan

Mustafa Said Aydogan

**N.** Hancerliogullari Nadia Alhazmi Nagwa Alamin

Naser Shari

Nasser Shubayr

Nawal Dayoub

Nesrin Alharthy

Nicola Magnavita

Nicola Mumoli

Nicola Troisi

Noorizan Abd Aziz

Nopporn Apiwattanakul

Noura Alqahtani

Nuha Alkhattabi

Nuri Bayram

**O**dd Martin Vallersnes

Ola A. Bakry

Omar Al Zahrani

Onur Fezvi Erer

Osama A. Samargandi

Osama B. Albasheer

**P**aolo Boffano

Paolo Cavoretto

Philip S. Barie

Pinar Korkmaz

Pinar Okyay

Polina Popova

Pradeep H. Navsaria

Q. E. Ali

Q. Ji

Qin Xiang Ng

Qintai Yang

Qun Wang

**R.** A. Gadelkareem

Rabie Soliman

Raghib Abusaris

Ram Das

Ram Samujh

Ramazan Akcan

Rami Abazid

Raymon Siu Ming Wong

Recep Akdur

Rehab Werida

Reinaldo Figueroa

Riwa Kishimoto

Roah Merdad

Ronald Oude Elferink

Rose Costa

Rudyard dos Santos Oliveira

**S**aad AlKhowaiter

Saad Alqahtani

Sabah Jastaneiah

Saddam Al Demour

Sakina Zerizer

Saleh Alanazi

Salih Ozgocmen

Salina Husain

Samah Abohamr*

Sameh Ahmed

Sameh Shehata

Samera AlBasri*

Sami Abu-Halaweh

Samy Kasem

Satyabhuwan Singh Netam

Sayed Abdulazeez

Selim Afsar

Selma Tosun

Sema Alacam

Serbulent Yigit

Seshadri Reddy Varikasuvu

Seyda Efsun Ozgunay

Shawn Thomas

Sheela Mathew

Sherif Abdelmonim

Shi-Yang Guan

Shoroq Altawalbeh

Shu-Qin yu

Sibtain Ahmed

Siraj Wali

Sirajudeen Shaik Alavudeen

Siva Reddy Challa

Sohaib Al-Khatib

Sohaib Shujaat

Sohail Ahmad

Stefan Opperman

Stefan Stortecky

Stephanie Baggio

Subhi Alghanem

Suhael Ahmed

Suheyla Abitagaoglu

Sultan Alamri

Sultan Alhowti

Sultan Ayesh Saghir

Sumayah Al Helali

Susan Busch

Svetlana Madjunkova

**T**ahir Hameed*

Taisuke Ito

Talal Alfaadhel

Tamer Abouelgreed

Tatjana Pekmezovic

Taymour Mostafa

Turki Albacker

Turki Al-Hussain

**U**lunay Kanatli

Umut Sabri Kasapoglu

**V.** Bedekovic

Veli Avci

Vikas Gupta

**W**afa I. Elhag

Waled Ahmed

Waleed Abdelfattah

Waleed Milaat

Waqas Sami

Wei Kang

**X**iang-jun Qiu

**Y**asemin Astandaa

Yasir Khayyat

Yasser Hadi

Yasser Samhan

Yassin Abdelsamad

Yoichi Nishimura

Yong Joo Kim

Yoshikatsu Nomura

Young Han Lee

Youssef M. Mosaad

Yusuf Aksu

**Z**aid Zoumot

Zainab A. Al Jufairi

Zakaria Al Habib

Zubair Syed

Zulqarnain Baloch
